# Effects of *Lespedeza Bicolor* Extract on Regulation of AMPK Associated Hepatic Lipid Metabolism in Type 2 Diabetic Mice

**DOI:** 10.3390/antiox8120599

**Published:** 2019-11-29

**Authors:** Younmi Kim, Heaji Lee, Sun Yeou Kim, Yunsook Lim

**Affiliations:** 1 Department of Food and Nutrition, Kyung Hee Univerity, 26 Kyung Hee-Daero, Dongdamun-Gu, Seoul 02447, Korea; younme_810@khu.ac.kr (Y.K.);; 2 Gachon Institute of Pharmaceutical Science, Gachon University, #191, Hambakmoero, Yeonsu-gu, Incheon 21936, Korea; sunnykim@gachon.ac.kr

**Keywords:** *Lespedeza bicolor*, type 2 diabetes, AMPK, lipid metabolism, inflammation, oxidative stress, fibrosis

## Abstract

*Lespedeza bicolor* (LB) is one of the ornamental plants used for the treatment of inflammation caused by oxidative damage. However, its beneficial effects on hyperglycemia-induced hepatic damage and the related molecular mechanisms remain unclear. We hypothesized that *Lespedeza bicolor* extract (LBE) would attenuate hyperglycemia-induced liver injury in type 2 diabetes mellitus (T2DM). Diabetes was induced by a low dosage of streptozotocin (STZ) injection (30 mg/kg) with a high fat diet in male C57BL/6J mice. LBE was administered orally at 100 mg/kg or 250 mg/kg for 12 weeks. LBE supplementation regardless of dosage ameliorated plasma levels of hemoglobin A1c (HbA1c) in diabetic mice. Moreover, both LBE supplementations upregulated AMP-activation kinase (AMPK), which may activate sirtuin1 (SIRT) associated pathway accompanied by decreased lipid synthesis at low dose of LBE supplementation. These changes were in part explained by reduced protein levels of oxidative stress (nuclear factor erythroid 2-related factor 2 (Nrf2) and catalase), inflammation (nuclear factor kappa B (NF-κB), interleukin-1β (IL-1β), interleukin-6 (IL-6), and nitric oxide synthases (iNOS)), and fibrosis (α-smooth muscle actin (α-SMA) and protein kinase C (PKC)) in diabetic liver. Taken together, LBE might be a potential nutraceutical to ameliorate hepatic damage by regulation of AMPK associated pathway via oxidative stress, inflammation, and fibrosis in T2DM.

## 1. Introduction

With around more than 500 million prevalent cases in 2018, type 2 diabetes mellitus (T2DM) is one of the most frequent metabolic syndromes in the world. In T2DM, insulin resistance (IR) caused by hyperglycemia leads to various diabetic complications [1,2,3]. As an insulin-sensitive tissue, the liver is susceptible to hyperglycemia-induced oxidative stress, which can cause hepatic damage [4,5].

Oxidative stress causes an imbalance between free radicals and antioxidants and reduces proliferation of mature hepatocytes [6,7,8,9]. As a result, chronic oxidative stress proliferates hepatic stellate cells (HSCs), which play a key role in the progression of hepatic fibrosis [10]. Nuclear factor erythroid 2-related factor 2 (Nrf2), a major transcription factor, regulates cellular resistance to oxidant exposure [11]. It modulates its downstream antioxidant defense mediators such as catalase, glutathione peroxidase (GPx), NAD(P)H dehydrogenase quinone 1 (NQO1), and superoxide dismutase (SOD) which eliminate excessive reactive oxygen species (ROS) [11]. Furthermore, oxidative stress triggers nuclear factor kappa B (NF-κB), which modulates various inflammatory mediators including interleukin-1β (IL-1β), interleukin-6 (IL-6), and tumor necrosis factor α (TNFα), causing chronic inflammation [9,11].

On the other hand, there is substantial evidence suggesting that adenosine monophosphate -activation kinase (AMPK) is dysregulated in metabolic syndrome such as obesity and T2DM [12]. More specifically, sirtuin1 (SIRT1) and AMPK have clinical relevance with regard to type 2 diabetes because of their effects on various cellular metabolism such as energy turnover, glucose metabolism, and lipotoxicity [12]. When the level of AMP is increased, AMPK is activated to maintain energy homeostasis. SIRT1 is an NAD+-dependent protein deacetylase which acts as a major regulator of energy homeostasis in response to nutrient availability. AMPK relies on SIRT1 activity to regulate lipid metabolism related pathway [12,13]. Moreover, AMPK downregulates lipogenesis related factors such as sterol regulatory element-binding protein1 (SREBP1), SREBP2, and fatty acid synthase (FAS) [12]. AMPK activation also decreases CAAT box/enhancer binding protein alpha (C/EBPα), which upregulates adipocyte differentiation [12,13,14]. Therefore, activation of AMPK associated pathway would be a therapeutic mechanism to reduce hepatic lipid accumulation in diabetes.

*Lespedeza bicolor* (LB) is a perennial deciduous shrub belonging to the leguminosae and is cultivated for ornamental purposes throughout Asia [15]. LB has been used traditionally for the treatment of inflammation. LB contains various compounds such as genistein, quercetin, daidzein, catechin, rutin, luteolin, and naringin [16]. These natural phytoconstituents affluent in *Lespedeza bicolor* extract (LBE) have been confirmed to exert antioxidants, decreasing the blood glucose level and anti-inflammatory activity. Especially, genistein, quercetin, and naringin have antioxidant activities such as electron donating and ROS scavenging activity [17]. Importantly, the previous study has shown that LBE ameliorated endothelial dysfunction induced methylglyoxal glucotoxicity in vitro [18]. Furthermore, LB attenuated methylglyoxal (MGO)-induced diabetic renal damage in vitro and in vivo [19]. These results suggest that LB had a potential for preventing or curing diabetic complications related to hyperglycemia.

However, no research has focused on the effect of LB on hyperglycemia-induced hepatic damage and its molecular mechanism. In this study, we hypothesized isoflavones and quercetin enriched LBE would ameliorative the effect on hyperglycemia-induced hepatic lipid metabolism by regulation of lipid metabolism in T2DM.

## 2. Materials and Methods

### 2.1. Preparation of Lespedeza Bicolor Extracts (LBE)

The aerial parts of LB were purchased from Jayeonchunsa Co. (Damyang, Korea). The preprocessing of LB was described in our previous research [18]. Briefly, LB was extracted with 70% ethanol at room temperature overnight. Afterwards, the extract was filtered, evaporated, and freeze-dried. The extract was dissolved in distilled water at a concentration of 100 and 250 mg/kg body weight (BW), respectively. The concentration of each stock solution was 25 mg/ml (low dosage of LBE, LL) and 62.5 mg/ml (high dosage of LBE, HL), respectively.

### 2.2. Animals and Experimental Design

C57BL/6J male (*n* = 40; 5-weeks-old) mice were provided (Raon Bio, Gyeonggi-do, Korea) and lodged in a room at 22 ± 1 °C, 50 ± 5% suitable humidity, and 12 h dark/light cycle. In a constant environment (12 h light/dark cycle, 21 ± 1 °C, and 50 ± 3% humidity), food and distilled water were supplied ad libitum. A randomly allocated diabetic group were fed with 40% kcal high-fat diet, while a non-diabetic control group (NC) was fed with 10% kcal control diet (AIN-93G). After 4 weeks, the diabetic group was intraperitoneally injected with 30 mg/kg body weight (BW) of streptozotocin (STZ) twice to induce diabetes [20]. The normal control mice were injected with only a citric acid buffer. One week after the second injection, mice with fasting blood glucose (FBG) levels higher than 140.4 mg/dl were included in the diabetic group. After induction of diabetes for 9 weeks, all mice were divided into 4 experimental groups (*n* = 10 per group) as follows: normal control, NC; diabetes mellitus control, DMC; low dosage of LBE, LL; high dosage of LBE, HL. The treatment groups were administrated with 100 mg/kg BW (LL) and 250 mg/kg BW (HL) by oral gavage every day for 12 weeks. LBE was freshly suspended in distilled water. At the same time, LBE untreated groups, the NC and DMC groups were treated with identical volumes of distilled water. During the treatment period, body weight, food intake, and fasting blood glucose (FBG) levels from the tail vein were measured once a week. At the end of treatment for 12 weeks, the animals were anesthetized by inhalation with diethyl ether (Duksan, Seoul, Korea). Blood sample was collected by heparin-coated (Sigma Aldrich, St. Louis, MO, USA) syringe from cardiac puncture and centrifuged at 845 g at 4 °C for 10 min to obtain plasma. The hepatic tissue was weighed and washed by saline. For protein extraction, part of the hepatic tissue was frozen in liquid nitrogen, and stored at −80 °C before experiments. Other parts of the hepatic tissue were fixed in 10% formaldehyde for paraffin embedding. All experiments were approved by Kyung Hee University for animal welfare (KHUASP(SE)-16-001) and were performed in accordance with the guidelines.

### 2.3. Hemoglobin A1c (HbA1c)

HbA1c levels were measured according to commercial reagent methods (Crystal Chem., Downers Grove, Elk Grove Village, IL, USA).

### 2.4. Plasma Glutamate Oxaloacetate Transaminase (GOT) and Glutamate Pyruvate Transaminase (GPT)

Plasma GOT and GPT were measured using commercial detection kits (Bio Clinical System, Gyeonggi-do, Korea).

### 2.5. Lipid Profile Analysis

Hepatic triglyceride (TG) and total cholesterol (TC) concentrations were measured using commercial kits (Bio-Clinical System, Gyeonggi-do, Korea) according to the manufacturer’s recommendation.

### 2.6. Histological Analysis

Hepatic tissue was fixed in 10% buffered formalin and embedded in paraffin wax. Histological sections (4 μm) of hepatic tissue were stained with hematoxylin and eosin (H&E) for conventional morphological evaluation using an optical microscope (Olympus BX51; Olympus Optical, Tokyo, Japan).

### 2.7. Western Blot Analysis

Hepatic tissue was homogenized in lysis buffer (20 mM Tris-Hcl, 150 mM NaCl, pH 7.5, 1% NP40, 0.5% Na-deoxycholate stock, 1mM ethylene diamineteraacetic acid (EDTA) and 0.1% sodium dodecyl sulfate (SDS)) and then centrifuged at 9000 g at 4 °C for 30 min. The supernatants were used for hepatic cytosol protein extract, the pelleted nuclei remnants were resuspended in a hypertonic buffer containing glycerol, 10 mM 4-(2-hydroxyethyl)-1-piperazineethanesulfonic acid (HEPES), 4 mM NaCl, 1 mM MgCl_2_, 500 mM EDTA, 1 mM dithiothreitol (DTT), phenylmethylsulfonyl fluoride (PMSF), 1 mM benzamidine, pepstatin, leupeptin, aprotinin, and distilled water. The lysed nuclei were stored at −80 °C until used for nuclear analysis. The hepatic extract was separated by 10% sodium dodecyl sulphate-polyacrylamide gel electrophoresis (SDS-PAGE) and transferred to a polyvinylidene fluoride (PVDF) membrane (Millipore, Billerica, MA, USA). The membranes were blocked with 3% bovine serum albumin (BSA) and incubated overnight at 4 °C with the primary antibodies: NF-κB, monocyte chemoattractant protein-1 (MCP-1), α-smooth muscle actin (α-SMA), catalase, C/EBPα, p-AMPK, AMPK, peroxisome proliferator-activated receptor-γ (PPARγ) (Cell Signaling Technology, Inc., Danvers, MA, USA, 1:500); Nrf2, SIRT1, nSREBP1, peroxisome proliferator-activated receptor-α (PPARα), FAS, GPx, NQO1, c-reactive protein (CRP), receptor AGE (RAGE) (Abcam, Cambridge, MA, USA, 1:1000); TNF-α, IL-1β, IL-6, MnSOD, transforming growth factor β (TGF-β), protein kinase C (PKC), CuZnSOD, peroxisome proliferator-activated receptor gamma coactivator 1-α (PGC1α), protein kinase C-βII (PKCβII), β-actin (Santa Cruz Biotechnology, Santa Cruz, CA, USA, 1:200); nitric oxide synthases (iNOS), cyclooxygenase-2 (COX2) (Stressgen, 1:1000); heme oxygenase-1 (HO-1), proliferating cell nuclear antigen (PCNA) (Enzolife science, 1:1000), 4-hydroxynonenal (4-HNE) (R&D system, Inc.). After washing by phosphate-buffered saline supplemented with Tween (PBS-T) three times, the membrane was then incubated with the relative secondary antibodies (Santa Cruz Biotechnology, Santa Cruz, CA, USA). After being washed an additional three times by PBS-T, the membranes were developed using the enhanced chemiluminescence (ECL) luminol reagent (Biorad, Hercules, CA, USA). The luminescent signal was recorded and quantified with the Syngene G box (Syngene, Cambridge, MA, USA).

### 2.8. Statistical Analysis

Results were presented as means ± SEM. The significance of difference was analyzed by one-way ANOVA followed by Tukey’s test. A probability level of *p* < 0.05 was considered statically significant. All statistical analysis used SPSS software (version 20.0 K for windows, Armonk, NY, USA) and Graphpad Prism (Version 5.0, San Diego, CA, USA).

## 3. Results

### 3.1. Effects of LBE Supplementation on Body Weight, Food Intake, and FBG Level in T2DM

After diabetes was induced, the body weight and FBG level of the DMC group was significantly higher compared to that in the NC group. However, LBE supplementation did not change body weight, food intake, and FBG levels in the diabetic mice (Table 1).

### 3.2. Effects of LBE Supplementation on Glycation Products in T2DM Mice

**Figure 1:**
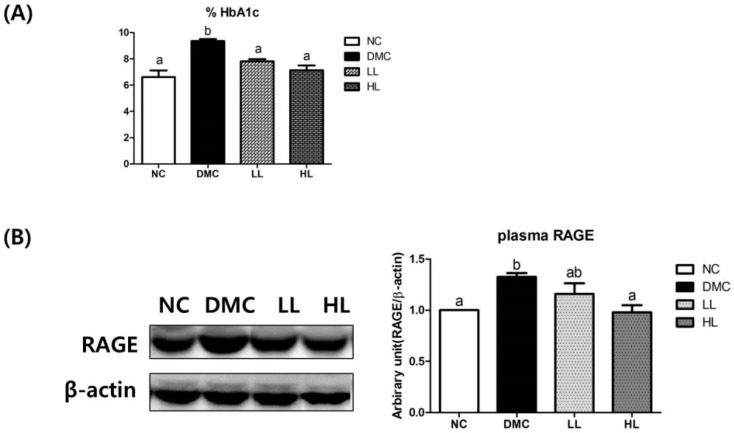
Effects of LBE supplementation on glycation products in T2DM mice. (**A**) %HbA1c and (**B**) plasma advanced glycation end products receptor (RAGE). Values are means ± SEM (*n* = 6). Mean values with different letters (^a^ and ^b^) were significantly different. (*p* < 0.05). NC: non-diabetic control group; DMC: diabetes mellitus control; LL: low dosage of LBE; HL: high dosage of LBE.

HbA1c and advanced glycation end products receptor (RAGE) expression in plasma were used to estimate advanced glycation end products (AGE) formation. As shown in Figure 1A, HbA1c was significantly higher in the DMC group than that in the NC group. However, LBE supplementation lowered the HbA1c level in the diabetic mice regardless of dose.

The protein levels of plasma RAGE in the DMC group were significantly higher compared to that in the NC group. The HL group showed significantly lower levels of RAGE than that in the DMC group. However, the protein level of RAGE in the LL group was not significantly different from that in the DMC group (Figure 1B).

### 3.3. Effects of LBE Supplementation on Plasma GOT and GPT in T2DM Mice

**Figure 2:**
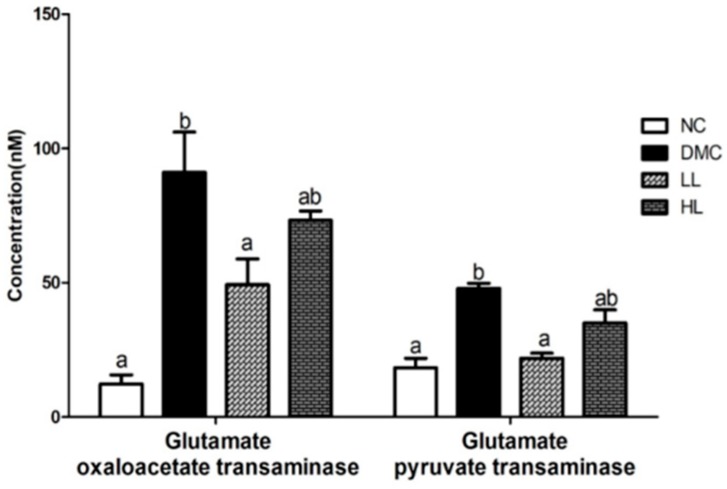
Effects of LBE supplementation on glutamate oxaloacetate transaminase (GOT) and glutamate pyruvate transaminase (GPT) in T2DM mice. Values are means ± SEM (*n* = 10). Mean values with different letters (^a^ and ^b^) were significantly different. (*p* < 0.05).

Plasma GOT and GPT levels were measured as biomarkers of liver injury. GOT and GPT levels were significantly higher in the DMC group than those in the NC group. Low dosage of LBE supplementation significantly lowered GOT and GPT levels compared to the DMC group whereas a high dosage of LBE supplementation did not (Figure 2).

### 3.4. Effects of LBE on Hepatic Morphology and Lipid Profiles in T2DM Mice

**Figure 3:**
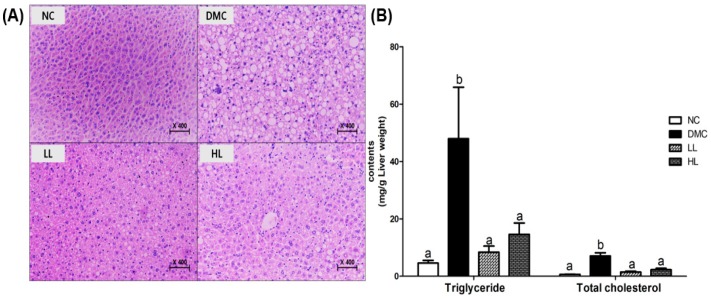
Effects of LBE supplementation on (**A**) hepatic morphology and (**B**) lipid profiles in T2DM mice. Levels of triglyceride (TG) and total cholesterol (TC) were measured in hepatic tissues. Values are means ± SEM (*n* = 6). Mean values with different letters (^a^ and ^b^) were significantly different. (*p* < 0.05).

Figure 3A shows the hepatic histology in each group. The white area estimated by fat deposition in the liver was increased in the DMC group compared to that in the NC group. However, in particular, the LL group showed a decrease in white areas compared to the DMC group. These findings could represent less fat deposition after the LBE treatment.

Moreover, TG and TC levels were significantly higher in the DMC group compared to those in the NC group. However, TG and TC levels in the LBE treatment groups were significantly lower than those in the DMC group (Figure 3B).

### 3.5. Effects of LBE Supplementation on Hepatic Protein Levels of Lipid Metabolism Related Markers in T2DM Mice

**Figure 4:**
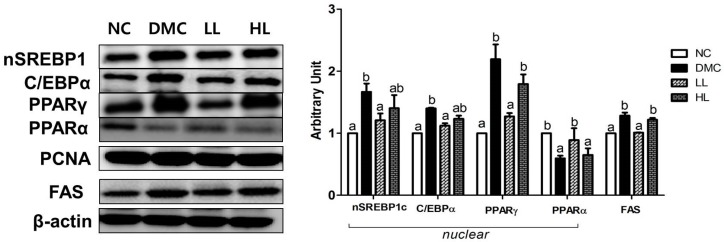
Effects of LBE supplementation on hepatic protein levels of lipid metabolism related markers: nuclear sterol regulatory element-binding protein1 (nSREBP1), CAAT box/enhancer binding protein alpha (C/EBPα), peroxisome proliferator-activated receptor-γ (PPARγ), peroxisome proliferator-activated receptor-α (PPARα), and fatty acid synthase (FAS) in T2DM mice. The hepatic protein was measured by Western blot. The bands show the intensity of the bands that were densitometrically measured and normalized to the band levels of proliferating cell nuclear antigen (PCNA) (nucleus) or β-actin (cytosol). Data are presented as means ± SEM (*n* = 6). Values with the same superscript letter (^a^ and ^b^) are not significantly different. (*p* < 0.05).

The protein levels of nSREBP1, C/EBPα, PPARγ, and FAS in the DMC group were significantly higher than those in the NC group. Only the LL group showed normalized lipid metabolism related markers compared to the DMC group. The protein level of PPARα in the DMC group was significantly lower than those in the NC group. The LL group showed a significantly higher level of PPARα than that in the DMC group (Figure 4). The protein level of FAS was increased in the DMC group compared to that of the NC group, but it was lowered by a low dose of LBE supplementation.

### 3.6. Effects of LBE Supplementation on Hepatic Protein Levels of Energy Metabolism Related Markers in T2DM Mice

**Figure 5:**
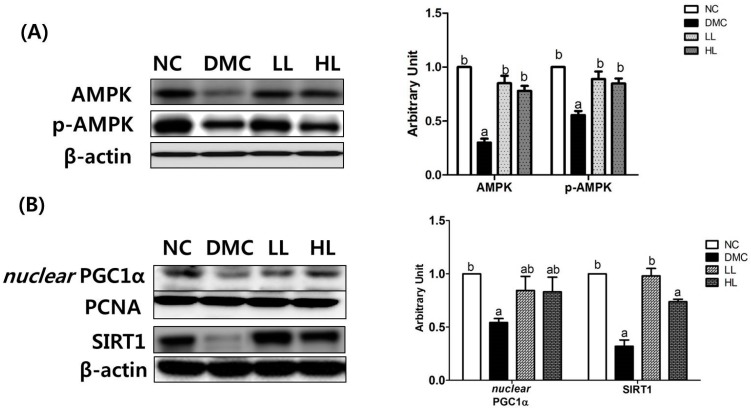
Effects of LBE supplementation on hepatic protein levels of energy metabolism related markers in T2DM mice. The hepatic protein was measured by Western blot. Representative band images of (**A**) adenosine monophosphate activation kinase (AMPK) phosphorylation and (**B**) nuclear peroxisome proliferator-activated receptor gamma coactivator 1-α (PGC1α) and Sirtuin1 (SIRT1) activation. The bands show the intensity of the bands that were densitometrically measured and normalized to the band levels of PCNA (nucleus) or β-actin (cytosol). Data are presented as means ± SEM (*n* = 6). Values with the same superscript letter (^a^ and ^b^) are not significantly different. (*p* < 0.05).

The protein levels of energy metabolism related markers including AMPK, p-AMPK, nuclear PGC1α, and SIRT1 were measured. The protein levels of AMPK, P-AMPK, *nuclear* PGC1α, and SIRT1 were decreased in the DMC group compared to those in the NC group. However, the protein levels of AMPK and p-AMPK in the LB treatment groups were significantly higher compared to those in the DMC group. The LL group showed a significantly higher level of SIRT1 than that in the DMC group. However, the protein level of PGC1α was not normalized by LBE supplementation (Figure 5).

### 3.7. Effects of LBE Supplementation on Plasma and Hepatic Protein Levels of Oxidative Stress Markers T2DM Mice

**Figure 6:**
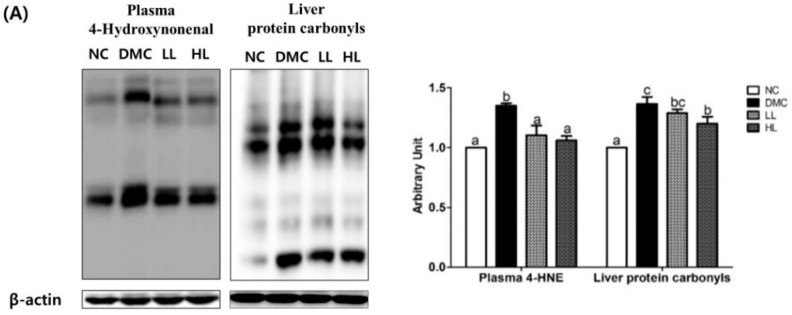

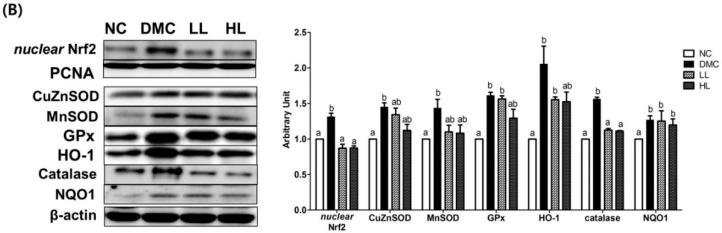
Effects of LBE supplementation on plasma and hepatic protein levels of oxidative stress markers in T2DM mice. (**A**) Plasma 4-hydroxynonenal (4-HNE) and liver protein carbonyls and (**B**) nuclear factor erythroid 2-related factor 2 (Nrf2) associated antioxidant defense markers: nuclear factor erythroid 2-related factor 2 (Nrf2), copper-zinc-superoxide dismutase (SOD), manganese superoxide dismutase (SOD), glutathione peroxidase (GPx), heme oxygenase-1 (HO-1), catalase, and NAD(P)H dehydrogenase quinone 1 (NQO1). The bands show the intensity of the bands that were densitometrically measured and normalized to the band levels of PCNA (nucleus) or β-actin (cytosol). Data are presented as means ± SEM (*n* = 6). Values with the same superscript letter (^a^ and ^b^) are not significantly different. (*p* < 0.05).

4-HNE and protein carbonyls were used as markers for oxidative stress in plasma (Figure 6A). The protein levels of plasma 4-HNE and protein carbonyls in the DMC group were significantly higher than those in the NC group. Both LL and HL groups showed significantly lower levels of 4-HNE than the DMC group. The level of protein carbonyls in the HL group was significantly lowered compared to that in the DMC group. The protein levels of nuclear Nrf2 and cytosolic CuZnSOD, MnSOD, HO-1, catalase, and NQO1 were significantly higher in the DMC group compared to those in the NC group. The protein levels of Nrf2 and catalase in the LBE supplementation groups were significantly lowered compared to those in the DMC group (Figure 6B). The protein levels of GPx were not different among the groups.

### 3.8. Effects of LBE Supplementation on Hepatic Protein Levels of Inflammatory Response Related Markers in T2DM Mice

**Figure 7:**
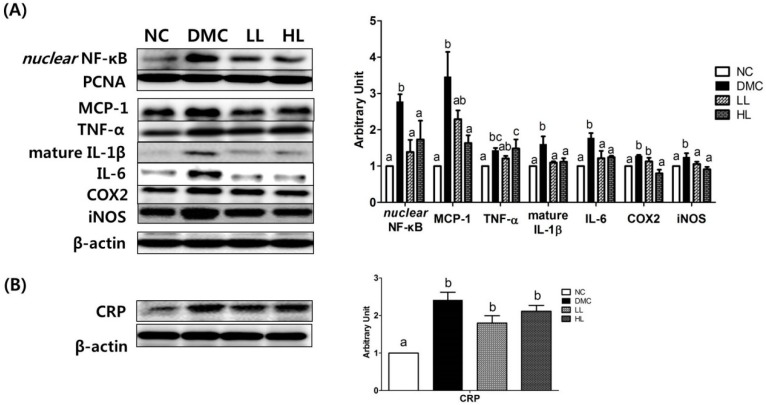
Effects of LBE supplementation on hepatic protein levels of inflammatory response related markers in T2DM mice. (**A**) Nuclear factor kappa B (NF-κB)-related markers: monocyte chemoattractant protein-1 (MCP-1), tumor necrosis factor α (TNFα), interleukin-1β (IL-1β), interleukin-6 (IL-6), cyclooxygenase-2 (COX2), and nitric oxide synthases (iNOS) and (**B**) inflammatory proteins: c-reactive protein (CRP). The hepatic protein was measured by Western blot. The bands show the intensity of the bands that were densitometrically measured and normalized to the band levels of PCNA (nucleus) or β-actin (cytosol). Data are presented as means ± SEM (*n* = 6). Values with the same superscript letter (^a^ and ^b^) are not significantly different. (*p* < 0.05).

The protein levels of inflammatory response related markers were measured by Western blot in hepatic tissue (Figure 7). The protein levels of nuclear factor kappa B (NF-κB) and its related inflammatory genes including TNF-α, IL-1β, IL-6, iNOS, MCP-1, and CRP were significantly higher in the DMC group than those in the NC group. However, the levels of NF-κB, IL-1β, IL-6, and iNOS in both LBE treated groups were significantly lowered compared to those in the DMC group. Furthermore, the protein levels of COX2 and MCP-1 were lowered in the HL group compared to the DMC group. The protein levels of TNF-α and CRP were not reduced in both LBE supplementation groups.

### 3.9. Effects of LBE Supplementation on Hepatic Fibrosis in T2DM Mice

**Figure 8:**
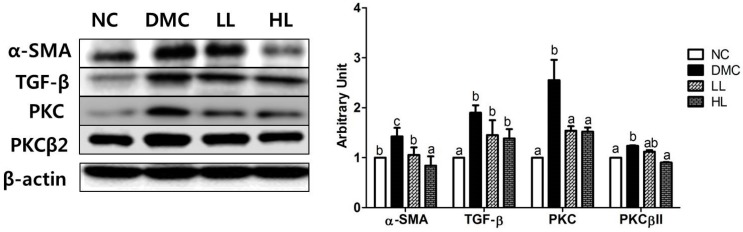
Effects of LBE supplementation on hepatic fibrosis markers: α-smooth muscle actin (α-SMA), transforming growth factor β (TGF-β), protein kinase C (PKC), and protein kinase C-βII (PKCβII) in T2DM mice. The hepatic protein was measured by Western blot. The bands show the intensity of the bands that were densitometrically measured and normalized to the band levels of β-actin (cytosol). Data are presented as means ± SEM (*n* = 6). Values with the same superscript letter (^a^, ^b^ and ^c^) are not significantly different. (*p* < 0.05).

The protein levels of fibrosis-related markers including α-SMA, TGF-β, PKC, and PKCβII were significantly higher in the DMC group compared to those in the NC group. The protein levels of α-SMA and PKC in both LBE treatment groups regardless of dose were significantly lower than those in the DMC group. Furthermore, the protein level of PKCβII only in the HL group was significantly lower than that in the DMC group (Figure 8). The protein level of TGF-β was not significantly reduced in both LB supplementation groups.

## 4. Discussion

In the present study, we investigated the effect of LBE on hyperglycemia-induced hepatic damage in diabetes. Consequently, the results demonstrated that LBE effectively attenuated hepatic damage by regulation of lipogenesis associated with oxidative stress, inflammation, and fibrosis in T2DM.

According to the HPLC analysis previously reported by our group, the concentration of genistein, daidzein, quercetin, and naringenin in LBE were determined as about 0.053 mg/g, 0.165 mg/g, 0.853 mg/g, and 0.08 mg/g, respectively [18]. These natural compounds showed antioxidant effects and exerted anti-diabetic and anti-lipogenic potentials in in vitro studies [21,22,23,24,25]. In the current study, a high dose of LBE supplementation reduced the levels of HbA1c along with RAGE, which was considered as an index of chronic hyperglycemic states [26], although a low dose of LBE treatment decreased only HbA1c, which is a more useful clinical biomarker of diabetes. Therefore, one can conclude that LBE is beneficial for attenuating the hyperglycemic condition in T2DM.

Hyperglycemia is a key contributor of hepatic damage in T2DM. We measured plasma levels of GOT and GPT which are sensitive clinical markers of hepatic damage [27]. Our data showed that a low dosage of LBE ameliorated hepatic damage by reducing GOT and GPT levels. Furthermore, LBE decreased hepatic fat droplets by reducing hepatic TG and TC levels in diabetes. These results are well in accordance with our histological observation of hepatic tissue. Therefore, LBE can be a potential nutrient attenuating hepatic lipid accumulation without extra hepatic burden.

We examined how LBE influences hyperglycemia-induced abnormal hepatic lipid metabolism. AMPK, which has a major role in lipid metabolism is known to decrease in diabetes [28]. Especially, AMPK downregulates the expression of SREBP1, which is a major transcription factor of fatty acid synthesis [29]. SREBP1 leads to an increase in the expression of lipogenic enzymes such as ACC and FAS [29]. Moreover, AMPK can change the NAD+/NADH ratio and accordingly stimulate SIRT1 expression in the hepatocyte [28,30]. SIRT1 activation also leads to an increase fatty acid oxidation via PPARα and PGC1α as well as a decrease in inflammatory response via NF-κB regulation [31]. Previous studies have shown that polyphenols in natural products activated AMPK-SIRT1 signaling pathway, which triggers lipogenesis and β-oxidation [31,32,33,34]. For the first time, the present study revealed that LBE treatment regardless of dosage increased the protein level of hepatic AMPK, which may increase NAD+ production by β-oxidation and lead to SIRT1 activation in T2DM mice.

In addition, LBE supplementation ameliorated hyperglycemia-induced oxidative stress in our study. Hyperglycemia-induced AGEs formation leads to RAGE production in the cell membrane [35]. Activated RAGE increases ROS, subsequently, leading to chronic oxidative stress [36]. As mentioned before, LBE supplementation at a high dose reduced the levels of RAGE expression in diabetic mice. A previous study also showed the ameliorative effects of LBE on MGO-induced RAGE expression in kidney tissue, which subsequently reduced AGE-RAGE interactions [37]. These results suggested that LBE reduced glycation products which can cause hepatic complications in diabetes. Furthermore, LBE has been known to have an antioxidant effect by scavenging nitrite in normal mice [38]. Our data demonstrated that the 4-HNE and protein carbonyls, representative biomarker of oxidative stress [39], were significantly increased in the DMC group compared to those in the NC. LBE supplementation regardless of dosage decreased the levels of 4-HNE but only a high dose of LBE treatment reduced protein carbonyl level in the diabetic mice. In addition, both LBE treatment reduced oxidative stress by regulation of Nrf2 and its downstream enzymes including catalase in diabetic liver.

Increased oxidative stress directly contributes to inflammation via activation of NF-κB, which regulates expression of inflammatory mediators [40]. In the current study, both LBE treatments significantly ameliorated NF-κB activation and its related inflammatory proteins including IL-1β, IL-6, and iNOS in diabetic mice and only a high dose of LBE treatment reduced levels of COX-2 and MCP-1, suggesting that LBE ameliorated hepatic hyper-inflammation related to NF-κB activation under diabetic condition. On the contrary, LBE treatment did not attenuate hepatic protein levels of CRP, which is not directly/indirectly regulated by NF-κB activation, in T2DM mice. A previous study also reported that LBE is capable of inhibiting NO production by inhibiting NF-κB in vitro [15]. As mentioned previously, genistein, quercetin, daidzein, and naringenin are affluent in LBE, and previous research showed that these compounds inhibited NF-κB activation along with decreased iNOS expression and NO production in vitro [41,42,43] and in vivo [44,45,46]. Therefore, it might be inferred that LBE supplementation might reduce hepatic oxidative stress along with NFκB associated inflammatory responses in T2DM.

Oxidative stress and inflammation resulting from chronic hyperglycemia also promote hepatic fibrosis in diabetes [46]. HSCs transform into proliferative and fibrogenic myofibroblasts, which express α-SMA in response to ROS [47]. Oxidative stress also induces the production of TGF-β and PKC which can cause cell death and activate collagen synthesis resulting in hepatic fibrosis [19,48]. The current study showed that LBE treatment regardless of dose attenuated hepatic protein levels of α-SMA and PKC II and only a high dose of LBE treatment reduced PKCβ level in T2DM mice. In the previous study, the level of increased fibrotic collagen in MGO-induced renal damage was attenuated by LBE in diabetic nephropathy [19]. Therefore, it can be concluded that LBE has an ameliorative effect on fibrosis related mediators along with reduced oxidative stress and inflammation in hyperglycemia-induced damaged tissue.

## 5. Conclusions

The present study demonstrated that LBE supplementation attenuated hyperglycemia-induced hepatic damage by regulation of AMPK associated lipogenesis in T2DM. Furthermore, LBE ameliorated hepatic oxidative stress, inflammation, and fibrosis although some molecular markers were selectively ameliorated at different treatment dosage of LBE in T2DM mice. Conclusively, LBE could be considered as a potential nutraceutical to ameliorate hyperglycemia-induced diabetic damage in T2DM.

## Figures and Tables

**Table 1:** Effects of *Lespedeza bicolor* extract (LBE) supplementation on body weight and food intake in type 2 diabetes (T2DM) mice.

Group	NC	DMC	LL	HL
Body weight (g)				
Before treatment	26.58 ± 1.35 ^a^	32.04 ± 3.29 ^b^	30.94 ± 2.40 ^b^	32.51 ± 4.13 ^b^
After treatment	30.42 ± 1.83 ^a^	43.93 ± 4.97 ^b^	39.02 ± 5.15 ^b^	40.34 ± 6.35 ^b^
Gain	3.85 ± 0.33 ^a^	8.89 ± 0.70 ^b^	8.09 ± 1.34 ^b^	7.82 ± 1.72 ^b^
Food intake (g/day)	2.40 ± 0.731 ^a^	3.36 ± 0.89 ^a,b^	3.56 ± 1.70 ^b^	4.16 ± 1.27 ^b^
Fasting blood glucose (FBG) (mg/dL)	122.2 ± 16.78 ^a^	173.8 ± 31.97 ^b^	135.6 ± 13.28 ^a,^^b^	170.8 ± 28.31 ^b^

Values are means ± SEM (*n* = 10). Mean values with different letters (^a^ and ^b^) were significantly different. (*p* < 0.05).

## Abbreviations

The following abbreviations are used in this manuscript:

AGE	Advanced glycation end products
AMPK	adenosine monophosphate activation kinase
AUC	Area under the curve
BSA	Bovine serum albumin
BW	Body weight
C/EBPα	CAAT box/enhancer binding protein alpha
DM	Diabetes mellitus
FAS	Fatty acid synthase
FBG	Fasting blood glucose
4-HNE	Four-hydroxynonenal
GOT	Glutamate oxaloacetate transaminase
GPT	Glutamate pyruvate transaminase
GPx	Glutathione peroxidase
H&E	Hematoxylin and eosin
HSCs	Hepatic stellate cells
IL-1β	Interleukin-1β
IL-6	Interleukin-6
LB	Lespedeza Bicolor
MGO	Methylglyoxal
NC	Normal control
NF-κB	nuclear factor kappa B
NQO1	NAD(P)H degydrogenase quinine 1
Nrf2	Nuclear factor erythroid2-related factor 2
OGTT	Oral glucose tolerance test
PVDF	Polyvinylidene fluoride
SIRT1	Sirtuin1
SOD	Superoxide dismutase
STZ	Streptozotocin
RAGE	Advanced glycation end products receptor
ROS	Reactive oxygen species
RNS	Reactive nitrogen species
SREBP1	Sterol regulatory element-binding protein 1
T2DM	Type 2 diabetes mellitus
TC	Total cholesterol
TG	Triglyceride
TNFα	Tumor necrosis factor α
